# CICU Workforce Surveys: Interpret With Caution

**DOI:** 10.1016/j.jacadv.2026.102920

**Published:** 2026-06-13

**Authors:** Amir Lotfi

**Affiliations:** Division of Cardiology, University of Massachusetts Chan Medical School-Baystate, Springfield, Massachusetts, USA

Papolos et al^1^ report results from the American College of Cardiology (ACC) Critical Care Cardiology Council (C^4^) workforce survey describing contemporary cardiac intensive care unit (CICU) organization and the evolving role of CICU cardiologists. The descriptive heterogeneity is useful for benchmarking. However, several methodological features should temper population-level interpretation, particularly any implication of temporal “shifts” toward high-intensity staffing.

First, representativeness is uncertain. Only 20% responded, and respondents were predominantly from academic centers.^1^ Because the unit of analysis is the individual clinician (with potential clustering by institution), reported proportions for “open,” “comanagement,” and “high-intensity” models should be interpreted as a description of respondents rather than national prevalence.

Second, measurement limitations matter. The instrument was novel and not formally validated, and several items allowed nonmutually exclusive selections or categorical ranges.^1^ In addition, “high-intensity” is defined broadly (any model in which a critical care medicine-boarded physician directly manages or comanages care), which can group operationally distinct structures (closed units, limited-hours comanagement, and dual-boarded CICU staffing).^1^^,^^2^ Without standardized operational details—coverage hours, admission authority, and consult/comanagement boundaries—comparisons across centers and with prior surveys are difficult. Relatedly, cross-sectional survey data cannot establish “growing trends” without longitudinal sampling.

Third, correlations between career satisfaction and work-life/burnout domains should be viewed as noncausal and potentially confounded; the constructs overlap conceptually, and missing responses were excluded from correlation analyses.^1^

These findings are best viewed as hypothesis-generating and most applicable to similar academic programs. Future work would be strengthened by institution-level probability sampling with weighting, prespecified staffing-model definitions, longitudinal measurement, and linkage to patient-centered outcomes and resource utilization.
